# The effects of *Eisenia fetida* and *Metaphire guillelmi* on the soil micro-food web in a microcosm experiment

**DOI:** 10.1371/journal.pone.0290282

**Published:** 2023-08-18

**Authors:** Xinli Niu, Yongfan Cheng, Xiaopei Feng, Wei Zhao, Xi Zhang, Mengjun Du, Yanfang Gu

**Affiliations:** School of Life Science, Henan University, Kaifeng, 475004, China; Feroze Gandhi Degree College, INDIA

## Abstract

Numerous studies have shown that the function of earthworms may depend on their ecotype and density, which affects how they impact soil microbial and nematode communities. However, it is unclear how different earthworm species and densities alter the composition of soil microbial and nematode communities and how these modifications impact the soil micro-food web. The structural equation model (SEM) is a more accurate tool for identifying the intricate relationships between various trophic levels in the soil micro-food webs than the widely used bivariate data analysis. In order to ascertain the effects of earthworm species, including epigeic earthworm *Eisenia fetida* and anecic earthworm *Metaphire guillelmi*, as well as varying densities on the composition of main microbial groups, soil nematodes and their relationships, a microcosm experiment was conducted in a greenhouse. After nine weeks of observation, compared with the control treatments, *Eisenia fetida* increased the biomasses of total microorganism and bacteria, whereas *Metaphire guillelmi* decreased the biomasses of total microorganism, bacteria, and gram-positive bacteria, but showed an increase in AMF biomass. Additionally, both two earthworm species decreased the abundance of total soil nematode, bacterivores, and omnivore-predators, which is in contrast with the control treatments. The SEM results indicated that the addition of *Eisenia fetida* at different densities had indirect negative effects on the abundance of omnivore-predators, as it significantly increased the content of soil organic carbon, ammonium nitrogen, and nitrate nitrogen. The bottom-up effects were found to be the dominant forces, which promoted bacterial-dominated decomposition channels. The addition of *Metaphire guillelmi* with different density had direct negative impact on bacterivores and fungivores. Moreover, it had indirect negative effects on omnivore-predators by altering the soil properties. The dominant forces were still the bottom-up effects. Our study suggests that different earthworm species have distinct mechanisms that affect the soil micro-food web in different ways.

## Introduction

In the agricultural ecosystem, earthworms make up the largest component of soil animal biomass and are commonly referred to as ecosystem engineers [1]. They have strong impact on soil physicochemical properties [2]. Recent study has addressed the effects of earthworms on plant production and microbial diversity [3,4]. The impact of earthworms on soil organisms is complex and numerous studies have shown that the mechanisms behind the effects are linked to the earthworm functional group, particularly the earthworm ecotype [1,3,5]. Epigeic earthworms reside in the little layer of above the mineral soil, where they frequently consume surface litter extensively [6]. In contrast, anecic earthworms inhabit permanent vertical burrows and feed on organic matter debris [7]. Additionally, different densities of earthworms produce various results [2,5,8]. Therefore, it is important to understand the impact of earthworm species and different densities of earthworms on the soil biological diversity and gain a better understanding the functions of soil biota in agroecosystems.

Soil microorganisms, including bacteria and fungi, microbivores like protozoa and nematodes, and predators such as nematodes, are all recognized as important components of the soil micro-food web [9]. Soil microorganisms, especially bacteria and fungi, play a crucial role in the transformation and cycling of soil nutrients [10,11]. Bacteria primarily absorb soluble and easily attainable substrates, whereas fungi commonly act as the primary decomposers of fresh plant litter. Soil nematodes and protozoa are the largest number predator of soil microorganisms at the second trophic level of soil micro-food web [12]. The highest trophic level in the soil micro-food web is occupied by omnivore-predator nematodes which feed on protozoa, soil nematodes, nematode eggs [13]. Distinct earthworm ecotypes can affect soil microorganisms, and soil nematodes through their feeding, burrowing, and casting activities in varying ways.

Previous studies demonstrated epigeic earthworms could either reduced [14] or increased soil microorganism biomass [15]. In addition, epigeic earthworms reduced the number of bacterivore nematodes [16], but had no effect on protozoa [17]. All of the preceding research suggested that epigeic earthworms can have a direct impact on microbes and nematodes, resulting in top-down effects. However, epigeic earthworms can also have indirect impact on soil microorganisms by changing the resource base. For instance, McLean et al. (2006) found that epigeic earthworms increased microbial biomass by conditioning litter matters and exerted bottom-up control [18]. As a result, when considering the effects of earthworms on the soil micro-food web, trophic interactions were critical. Only a few studies have examined the interactions of epigeic earthworms with a few trophic groups of the soil micro-food web. Epigeic earthworms decreased the number of nematodes and affected the number of protozoa differently depending on the species of earthworm and protozoa [19]. Simultaneously, the effects of epigeic earthworms on soil microorganisms varies depending on the density of earthworms present. As the density of the epigeic *Lumbricus rubellus* increased, the total nematode density decreased compared to the control treatments [20]. Moderate density of epigeic earthworms increased microarthropod densities, whereas high density of earthworms decreased them [8].

Compared with epigeic earthworms, anecic earthworms have longer intestines, a simpler and fewer typhlosole, extended transportation, and increased contractility [21]. These factors might lead to varying effects of anecic earthworms on soil organisms. The anecic earthworm species *Pheretima guillelmi* decreased the total biomass of soil microorganisms [22], reduced nematode density [23], and either decreased [24] or increased the density of protozoa [23]. However, most of the previous studies on the effects of anecic earthworms on soil organisms have focused solely on a single taxon, rather than examining trophic interactions within the soil micro-food web. To our knowledge, there have been very few studies of anecic earthworms on the soil micro-food web. It has been observed that high density earthworms stimulated nematode populations but have a negative impact on collembolans through bioturbation and mucus excretion, indicating that earthworms altered the bottom-up impacts on soil organisms [25]. The presence of anecic earthworms strengthened the biotic associations of soil microbial and microfaunal communities [3]. Anecic earthworms had little influence on the structure and interactions of the soil micro-food web [26].

Earthworms are known to have an impact on the soil micro-food web directly through predation, as well as indirectly through the modification of soil abiotic factors via bioturbation [27]. The impact of ecosystem engineers on the structure of soil micro-food web is rarely discussed [28,29]. It is unclear whether the presence of earthworms primarily mediates bottom-up forces, top-down effects, or both simultaneously. Our objective was to evaluate how the presence of two “ecosystem engineer”, the epigeic earthworm *Eisenia fetida*, and the anecic earthworms *Metphiren guillelmi* affected the biotic, abiotic, and non-trophic interactions among soil organisms, respectively. We hypothesized that (1): in the presence of epigeic and anecic earthworms, bottom-up effects may be more important than top-down forces; (2) different species of earthworms have diverse impact mechanisms on the soil micro-food web.

## Materials and methods

### Experiment soil

A mineral soil with loamy sandy texture, located 0–20 cm below the surface, was collected from an experiment field at Henan University where winter wheat (*Triticum aestivum*) and maize (*Zea mays*) were being rotated. Stones, extensive roots, and macro-arthropods were manually removed. The soil was dried in the air after being sieved with 2 mm mesh. The soil had a bulk density of 1.98g/cm^3^ and consisted of 10–11% clay, 52–58% sand, and 30%-40% loam. The original soil sample was retained for analysis of its physical and chemical characteristics. The initial soil contained approximately 0.52g/kg total nitrogen, 5.66g/kg organic carbon, 14.27 g/kg total carbon (C/N ratio 10.88), 13.56 mg/kg nitrate nitrogen, 4.18mg/kg ammonium nitrogen, and had a pH of 8.12.

### Experimental earthworms

We inoculated two species of earthworms, the epigeic *Eisenia fetida* and the anecic *Metaphire guillelmi* in the microcosm experiment. The *Eisenia fetida* and *Metaphire guillelmi* were bought from a worm farm in Jurong city, Jiangsu Province. They were cultured in a plastics box (length × width × height: 75 cm × 50 cm × 50 cm, soil moisture 60%) contained with maize straw and the experimental soil, and were raised in a dark environment in the laboratory (25°C, relative humidity 60%). The small *Eisenia fetida* inhabits topsoil and feeds on organically rich food, consuming only a small amount soil. However, the *Metaphire guillelmi* is a soil-dwelling species and extensively distributed in China. In terms of ecological traits, it differs significantly from *Eisenia fetida*. Large *Metaphire guillelmi* lives in semi-permanent vertical deep burrows where they ingest the soil particles to assimilate organic matter [30].

### Experimental plants

Zhoumai 18 cultivars were selected as winter wheat seeds. We got the seeds from Plant Genetic Resources and Genetic Engineering Laboratory, Henan University. They were sterilized with sodium hypochlorite (1%) for ten minutes, then rinsed repeatedly with distilled water, and sown on wet paper in Petri dishes. Finally, they were placed in a climate chamber (14 h/10 h, light/dark, 25°C).

### Earthworm food

The food for the earthworms was obtained from the maize straw that had been harvested in the experimental field of the school of life sciences, Henan University. The maize straw was sundried and milled (ball mill) to pass through a 2 mm diameter mesh before use. The maize straw contained 333.06g/kg of total carbon, total nitrogen 9.21g/kg, total phosphorus 1.86g/kg, and 36.16 the ratio of carbon to nitrogen. In accordance with the earthworm’ organic matter consumption, 0.12 kg of maize straw (equivalent to 3% of the dry soil weight in each pot) was added into each pot, and mixed thoroughly with the soil.

### Experimental design

The experiment, which included the control and earthworm addition treatments was carried out in a greenhouse at Henan University in China. The control treatments did not involve the inoculation of any earthworms. The earthworm addition treatments were divided based on the addition of two species of earthworm. The earthworm densities were further categorized into three groups: addition of 1, 2, and 4 earthworms per pot, respectively. The density of earthworm addition in our microcosm experiment was 216 individuals/m^2^, which was observed in a winter wheat field in the Huang-Huai-Hai plain [31]. Meanwhile, we set the different density earthworm additions at 0, 1, 2 and 4 individuals per pot, which corresponds to 0, 54, 108, and 216 individuals / m^2^. Each treatment had twenty replicates, resulting in 140 microcosms (140 pots).

The diameter and height of the pots are 15 cm and 35 cm, respectively. Gauzes with a 1mm bore diameter was placed at the bottom of each pot to prevent earthworms from escaping. A total of 560 kg soil (dry weight) and 16.8 kg maize powder (earthworm food) were thoroughly mixed and divided into 140 plastic pots. Each pot contained 4 kg of soil and 0.12 kg of maize straw. Before the experiment, the gut contents of 280 earthworms, including 140 *Eisenia fetida* and 140 *Metaphire guillelmi* individuals, were emptied on moistened tissue paper for 24 h at room temperature. Then, we added 140 *Eisenia fetida* earthworm individuals into 60 pots, corresponding to 1, 2, and 4 individuals per pot, respectively. Each of the twenty pots contained 1 earthworm individual, while another twenty pots had 2 earthworm individuals, and the remaining twenty pots had 4 earthworm individuals. The addition method for 140 *Metaphire guillelmi* earthworms was similar to that of 140 *Eisenia fetida* earthworm individuals in that they were added into 60 pots with 1, 2, and 4 individuals per pot, respectively. Earthworms were placed on the surface of the soil in pots and actively dug into it. Earthworms that were burrowing into the soil were carefully examined after they were inoculated. If necessary, earthworms with high activity could be used to replace earthworms with low activity. To prevent earthworms from escaping from the experimental pots, 140 gauzes with a diameter of 25 cm and a height of 30 cm were wrapped around the edge of pots and fixed on a wireframe. After a week, germinated winter wheat plant seeds (one cotyledon) were transplanted into each pot. Every week, the 140 microcosms were redistributed at random in the greenhouse. The pot experiment began on 8^th^, November in 2021. During the experiment, no fertilizers were used, and any weed seedlings that germinated from the soil were manually removed. All of the pots were maintained at 70% soil field capacity and watered with tap water every two days using the weight method. Winter wheat was grown in greenhouses for nine weeks (relative humidity 60–85%, temperature 25°C, light intensity 600 μmol / m^2^ / s, and photoperiod 14 h).

### Harvesting plants, two species earthworms, and soil sampling

After nine weeks, all of winter wheat plants were cut at ground level and separated into two parts: aboveground and belowground. The belowground parts were washed separately, and then aboveground and belowground dry weights of the winter wheat were determined after being dried for 72 h at 70°C.

Earthworms were collected, rinsed, wiped with absorbent paper, and then weighted. We collected 220 earthworm individuals. Five replicates of each treatment were excluded because the number of earthworms at the end of the experiment were not equal to the number at the start of the experiment in those treatments. The harvested earthworms were transferred into enamel plates simultaneously, and a certain amount of clear water was added to each plate, followed by a slow addition of 95% alcohol until the clear water in the plate became a 10% alcohol solution. After two hours, we measured the body length (cm) and body width (cm) of these earthworms. Subsequently, we performed dissections and measured their physical characteristics such as gizzard diameter (mm), foregut (cm), midgut (cm), and hindgut (cm).

We thoroughly mixed the soil in each pot after harvesting the winter wheat and earthworms. Three soil samples were taken from each pot. These three samples were then combined to form one composite sample. In total, 105 composite samples were collected.

### Soil analysis

The analyzed soil properties included total carbon (TC), total nitrogen (TN), total phosphorus (TP), organic carbon (SOC), available phosphorus (available P), nitrate nitrogen (NO_3_^-^N), ammonium nitrogen (NH_4_^+^N), and soil pH (H_2_O). TC, TN, and SOC was analyzed using an element analyzer (Vario MACRO cube, Elementar Inc., Germany). The molybdenum blue colorimetric method was used to determine TP. Soil available P was determined by molybdenum antimony colorimetric, NaOH melting, and 0.5mol/L NaHCO_3_ extraction, respectively. NO_3_^-^N and NH_4_^+^N were measured using a Smart Chem 200 Discrete Auto Analyzer (AMS Systea, Italy) (Wei, Zheng, Li, Lü, Yu et al. 2012). Soil pH was measured in a 1:2.5 soil-distilled H_2_O suspension using a glass electrode (Sartorius PB-10).

### PLFA analysis

The composition of the soil microbial community was determined using phospholipid fatty acid (PLFA) analysis based on the methods of Bossio et al. [32]. PLFA was extracted from 8 g freeze-dried with a single-phase chloroform-methanol-citrate buffer (1: 2: 0.8) in 23 ml extraction mixture containing chloroform: phosphate: buffer (1: 2: 0.8 v/v/v). The concentration of each PLFA was calculated based on a c19:0 internal standard, and the abundance of the individual fatty acids was identified as nmol lipid per gram of dry soil. Gram-negative bacteria biomass was estimated based on the sum of 16:1ω7c, 16:1ω9c, 17:0cy, 17:1ω8c, 18:1ω5c, 18:1ω7c and 19:0cyω8c, and Gram-positive biomass was estimated based on the sum of 14:0i, 15:0i, 15:0a, 16:0i, 17:0i and 17:0a [33,34]. The sum of the gram-negative bacteria, gram-positive bacteria and non-specific bacteria (14:0, 15:0, 16:0, 17:0 and 18:0) was expressed as the total bacteria biomass. 18:1ω9c and 18:2ω6c represented the biomass of total fungi [35,36]. The biomass of Arbuscular mycorrhizal fungi was estimated as the sum of 16:1w5c. The biomass of actinomyces was determined as the sum of 10Me16:0, 10Me17:0, and 10Me18:0, and protozoa were identified by the PLFA biomarker 20:2ω6c, 20:3ω6c, and 20:4ω6c [37].

### Nematode analysis

For each composite soil sample from each pot, nematodes were extracted from 100 g of fresh soil using the modified Baermann method [38]. Another 100 g fresh soil was used to determine the soil water content. The extracted nematodes were preserved in TAF fixation (40% formaldehyde 7 ml, triethanolamine 2 ml, and distilled water 91 ml). Nematode abundance was expressed as individuals per 100 g of dry soil. After counting the total number of nematodes, 100 nematode individuals from each sample were identified to the genus level according to Bongers [39] by using an optical microscope (Motic, BA210, Motic Corporation). All specimens in samples with fewer than 100 nematodes were identified. The soil nematodes were divided into four trophic groups: bacterivores (Ba), fungivores (Fu), plant parasites (Pp), and omnivores-predators (Om), each with their own colonizer-persister (cp) groups [39,40].

### Statistical analysis

We analyzed only the samples in which the number of earthworms found matched the number introduced. The paired t-test was used to compare the amount of earthworm biomass at the start and end of our experiment. Prior to statistical analysis, microorganism biomass and nematode abundances were ln(x+1) transformed to ensure data normality. The linear mixed model was used to investigate the effects of earthworm species, different earthworm density, and the nested densities of earthworm species on soil properties, main microbial groups, and soil nematode community. Earthworm species and earthworm density were assigned as fixed factors in the model, while replicates were assigned as the random factors, and earthworm biomasses at the start of experiment were used as covariant. A difference at *P* < 0.05 was considered to be statistically significant. All statistical analysis was performed using SPSS v.19.0 (SPSS Inc., Chicago. IL). Principal component analysis (PCA) and redundancy analysis (RDA) were performed to explore the composition of the soil biotic community based on the relative abundances of PLFAs and nematode’ data and the relationship between soil biota and environmental parameters using CANOCO software, version 4.5.

Structural equation modeling (SEM) is a multivariate statistical method for testing hypothesized complex path-relationship networks and providing scientific answers [41,42]. SEM was used in the study to determine the potential mechanisms of the food chain under different densities of each earthworm species. Based on a literature review and our observations of predators in the soil micro-food web, we constructed a priori model including all possible relationships among predators. SOC, NO_3_^-^N, and NH_4_^+^N were treated as the indicators of soil properties, and the biomass of bacteria, and fungi of soil microorganisms, and all nematode trophic group abundance were treated as the indicators of observed variables. Before testing the model, all bivariate relationships were examined for signs of nonlinearities as well as the normality for heteroscedasticity. The analysis was conducted using AMOS 7.0 software [43]. The χ^2^ value and the associated *P*-value were used to judge the goodness of fit of the model to the date. The comparative fit index (CFI), goodness-of-fit (GFI) and root square mean error of approximation (RMSEM) were used to evaluate the model fit. We selected the model that fits best our data.

## Results

### Biomasses of two earthworm species, and the biomasses of winter wheat

The initial weight of two species of earthworms at the beginning and end of the experiment is shown in Table 1. At the end of the experiment, paired-samples t tests indicated that *Eisenia fetida* biomasses increased by 8.51%, 8.24% and 6.25% under added 1, 2, and 4 density treatments, respectively, and *Metaphire guillelmi* biomasses increased by 3.21%, and 1.81% under added 1, and 2 density treatments, respectively, when compared to the biomasses of each earthworm species at the beginning of the experiment (Table 1).

**Table 1:** The total biomasses (g) (means ± SE) and the results of paired-t test of *Eisenia fetida* and *Metaphire guillelmi* in each pot at the beginning and end of the experiment (n = 15, T-test).

Earthworm species	Treatments	Earthworm biomasses at the beginning of experiment	Earthworm biomass at the end of experiment	df	t	*P*
*Eisenia fetida*	C	0	0	4	0	0
	+1E1	0.44±0.01	0.47±0.01	4	-3.25	0.006
	+2E1	0.84±0.01	0.86±0.01	4	-2.28	0.039
	+4E1	1.76±0.02	1.79±0.02	4	-2.99	0.010
*Metaphire guillelmi*	C	0	0	4	0	0
	+1E2	3.85±0.10	4.10±0.08	4	-4.13	0.001
	+2E2	7.19±0.10	8.07±0.17	4	-6.41	0.000
	+4E2	14.13±0.12	15.19±0.22	4	-6.28	0.000

C, control (no added earthworm); +1E1, +2E1, and +4E1 represents added 1, 2, and 4 *Eisenia fetida* earthworm individuals, respectively; +1E2, +2E2, and +4E2 represents added 1, 2, and 4 *Metaphire guillelmi* earthworm individuals, respectively.

**Fig 1:**
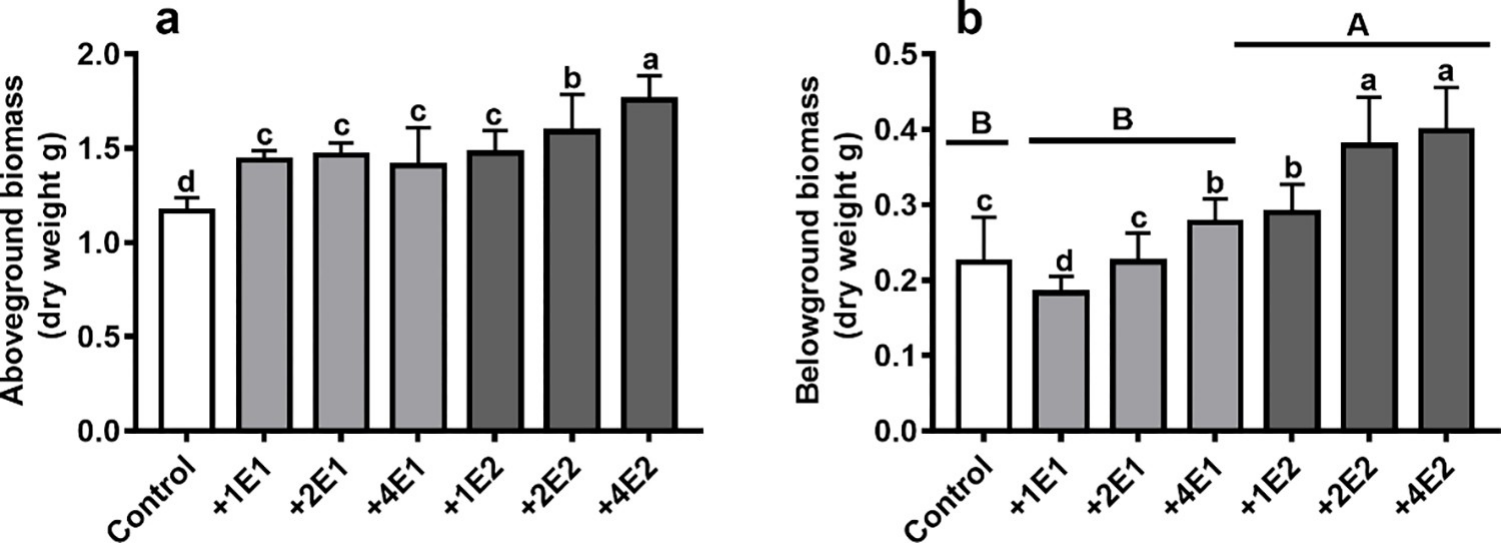
The aboveground and belowground biomasses (dry weight g) of winter wheat under two earthworm species (*Eisenia fetida*, *Metaphire guillelmi*), and the different density of *Eisenia fetida* and *Metaphire guillelmi* treatments. +1E1, +2E1, and +4E1 represents added 1, 2, and 4 *Eisenia fetida* earthworm individuals, respectively; +1E2, +2E2, and +4E2 represents added 1, 2, and 4 *Metaphire guillelmi* earthworm individuals, respectively. Different uppercase letters indicate significant difference in different earthworm species. Different lowercase letters indicate significant difference in different density under each earthworm species, relative to the control treatments (replicates = 15).

The physical indices of two earthworm species were shown in **Table 2**. Compared with *Eisenia fetida*, *Metaphire guillelmi* had a longer and wider body, a bigger gizzard, and longer foregut, midgut and hindgut. Earthworm species significantly affected the belowground biomasses of winter wheat **(Table 3)**. Compared with the control treatments, the presence of *Metaphire guillelmi* dramatically increased the belowground biomasses of winter wheat (Fig 1B, Table 3). Different earthworm density significantly affected the aboveground and belowground biomasses of winter wheat (Table 3, Fig 1A and 1B).

**Table 2:** The physical indices (Mean ± se) of two earthworm species including *Eisenia fetida* (105 individuals) and *Metaphire guillelmi* (105 individuals) at the end of the experiment.

Earthworm species	Body length (cm)	Body width (cm)	Gizzard diameter (mm)	Foregut (cm)	Midgut (cm)	Hindgut (cm)
*Eisenia fetida*	6.81±0.05b	0.33±0.00b	1.91±0.02b	1.82±0.01b	4.28±0.03b	0.57±0.01b
*Metaphire guillelmi*	11.90±0.04a	0.63±0.01a	3.34±0.02a	2.67±0.02a	7.72±0.02a	0.99±0.01a

Different lowercase letters represent significant differences in earthworm physical indices between *Eisenia fetida* and *Metaphire guillelmi*, as determined by independent-sample t-test, *P* < 0.05.

**Table 3:** Results of linear mixed models examining effects of earthworm species, the different earthworm density addition, and the nested density of earthworm species on soil physical and chemical properties.

Soil physical-chemical properties	Earthworm species			The different earthworm density addition			Earthworm species (earthworm density)		
	df	F	*P*	df	F	*P*	df	F	*P*
AG(g)	1	9.69	1.000	2	5.01	**0.009**	2	11.36	**0.000**
BG(g)	1	14.62	**0.000**	2	14.10	**0.000**	2	3.79	**0.026**
TC	1	0.52	0.471	2	1.24	0.295	2	1.15	0.322
TN	1	7.26	0.158	2	6.80	**0.002**	2	3.06	0.053
TP	1	0.10	0.757	2	0.48	0.619	2	0.16	0.854
SOC	1	0.28	0.609	2	18.80	**0.000**	2	0.81	0.450
Available P	1	0.04	0.851	2	0.45	0.655	2	1.23	0.297
NH3+ N	1	3.76	0.057	2	5.90	**0.004**	2	1.84	0.165
NH4+N	1	0.84	0.374	2	37.15	**0.000**	2	0.18	0.833
pH	1	17.68	**0.000**	2	2.20	0.116	2	3.60	**0.031**

Total carbon (TC), total nitrogen (TN), total phosphorus (TP), organic carbon (SOC), available phosphorus (available P), nitrate nitrogen (NO_3_^-^N), and ammonium nitrogen (NH_4_^+^N). AG, the aboveground biomasses of winter wheat; BG, the belowground biomasses of winter wheat.

### Soil physical and chemical properties

Earthworm species significantly affected soil pH (Table 3). Compared with the control treatments, *Metaphire guillelmi* addition significantly increased soil pH. Different earthworm density significantly affected soil TN, SOC, NO_3_^-^N and NH_4_^+^N content (Tables 3 and **4**). Compared with the control treatments, the content of SOC, NO_3_^-^N, and NH_4_^+^N increased with the increasing density of added *Eisenia fetida* or *Metaphire guillelmi* earthworms (Tables 3 and 4). The highest SOC, NO_3_^-^N, and NH_4_^+^N content was found both at added 4 *Eisenia fetida* or *Metaphire guillelmi* earthworm treatments (Table 4).

**Table 4:** Soil physical and chemical properties (means ± SE) under the different density *Eisenia fetida* and *Metaphire guillelmi* addition treatments (n = 15).

Earthworm species	Treatments	TC (g/kg)	TN (g/kg)	TP (g/kg)	SOC (g/kg)	Available P(mg/kg)	NO_3_^-^N (mg/kg)	NH_4_^+^N (mg/kg)	pH
*Eisenia* *fetida*	C	14.94±0.09	0.51±0.01c	0.67±0.01	5.26±0.08d	19.23±0.20	13.25±0.08c	4.06±0.07d	8.06±0.01c
	+1E	15.57±0.03	0.56±0.00b	0.67±0.01	5.93±0.08c	19.81±0.17	14.44±0.07b	4.50±0.08c	8.09±0.01
	+2E	15.33±0.07	0.61±0.01a	0.69±0.00	6.32±0.09b	19.06±0.20	15.06±0.12a	4.88±0.04b	8.08±0.01
	+4E	15.14±0.08	0.64±0.02a	0.70±0.00	6.61±0.10a	19.42±0.22	15.08±0.17a	5.16±0.09a	8.08±0.01
*Metaphire* *guillelmi*	+1E	15.16±0.10	0.55±0.01a	0.68±0.00	5.98±0.07c	19.29±0.19	14.73±0.18b	4.56±0.03c	8.12±0.02b
	+2E	15.09±0.03	0.58±0.01a	0.68±0.00	6.07±0.09bc	19.48±0.16	15.03±0.19ab	4.84±0.03b	8.17±0.01a
	+4E	15.24±0.15	0.57±0.01a	0.69±0.00	6.47±0.10a	19.17±0.16	15.35±0.18a	5.10±0.07a	8.15±0.01ab

Total carbon (TC), total nitrogen (TN), total phosphorus (TP), organic carbon (SOC), available phosphorus (available P), nitrate nitrogen (NO_3_^-^N), and ammonium nitrogen (NH_4_^+^N). C, control (no added earthworm); +1E1, +2E1, and +4E1 represents added 1, 2, and 4 *Eisenia fetida* earthworm individuals, respectively; +1E2, +2E2, and +4E2 represents added 1, 2, and 4 *Metaphire guillelmi* earthworm individuals, respectively.

### Soil microorganism communities

**Fig 2:**
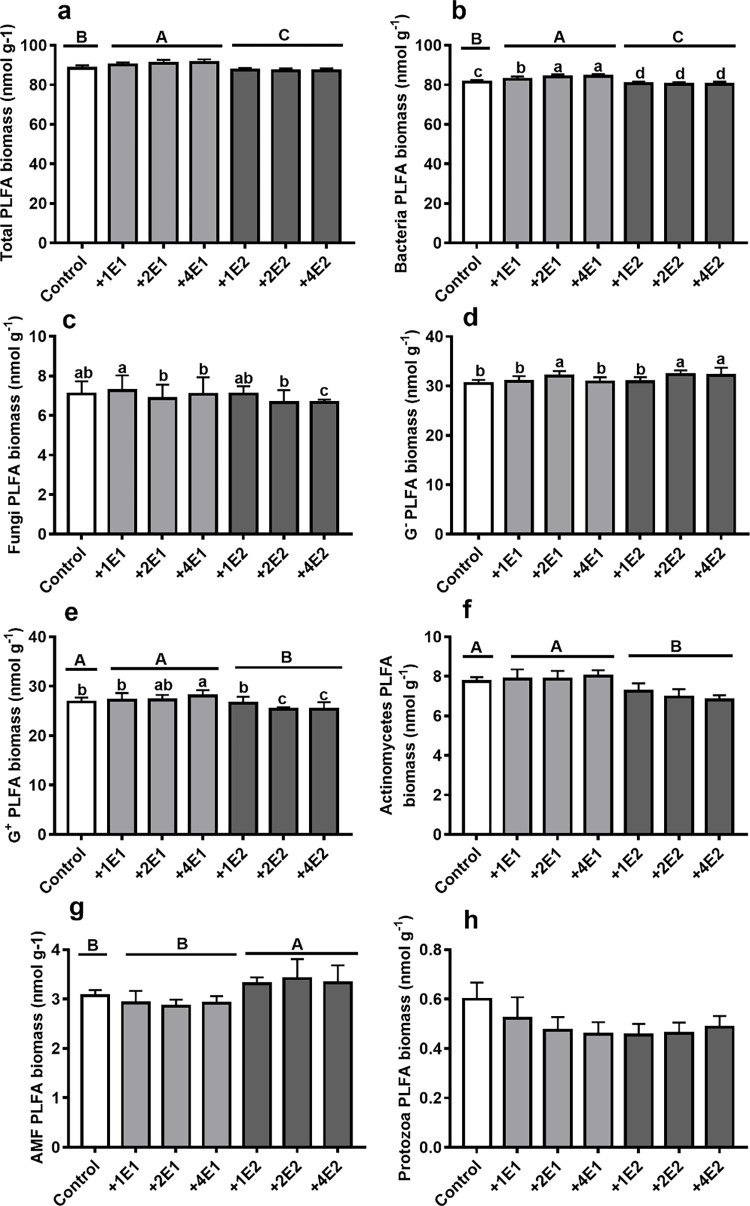
The biomass of soil total microorganism (a), bacteria (b), fungi (c), gram-negative bacteria (d), gram-positive bacteria (e), actinomycetes (f), AMF (arbuscular mycorrhizal fungi) (g), and protozoa (h) under two earthworm species (*Eisenia fetida*, *Metaphire guillelmi*), and the different density of *Eisenia fetida* and *Metaphire guillelmi* treatments. C, control (no added earthworm); +1E1, +2E1, and +4E1 represents added 1, 2, and 4 *Eisenia fetida* earthworm individuals, respectively; +1E2, +2E2, and +4E2 represents added 1, 2, and 4 *Metaphire guillelmi* earthworm individuals, respectively. Different uppercase letters indicate significant difference in different earthworm species. Different lowercase letters indicate significant difference in different density under each earthworm species, relative to the control treatments (replicates = 15).

Earthworm species had significant impact on the biomasses of total microorganism, bacteria, gram-positive bacteria, actinomycete and AMF **(Table 5)**. In comparison to the control treatments, *Eisenia fetida* significantly increased the biomasses of total soil microorganism, and bacteria, whereas *Metaphire guillelmi* decreased them (Fig 2A and 2B, Table 5). Compared with the added *Eisenia fetida* treatments, *Metaphire guillelmi* dramatically decreased gram-positive bacteria biomass (Fig 2E, Table 5), and actinomycete biomass (Fig 2F, Table 5), whereas increased AMF biomass (Fig 2G, Table 5). Different earthworm density significantly affected the biomasses of bacteria (Fig 2B), gram-negative bacteria (Fig 2D), and marginally affected fungi (Fig 2C) (*P* = 0.054< 0.10) and gram-positive bacteria (Fig 2E) (*P* = 0.070 < 0.10).

**Table 5:** Results of linear mixed models examining effects of earthworm species, the different earthworm density addition, and the nested density of earthworm species on soil microorganism biomasses including total microorganism, bacteria, fungi, the ratio of gram-positive bacteria to gram-negative bacteria, gram-negative bacteria, gram-positive bacteria, the ratio of gram-positive to gram-negative bacteria, actinomycete, arbuscular mycorrhizal fungi (AMF), protozoa.

Soil microorganism index	Earthworm species		The different earthworm density addition		Earthworm species (earthworm density)	
	F	*P*	F	*P*	F	*P*
Total soil microbial biomass	46.69	**0.000**	1.40	0.251	4.77	**0.011**
Bacteria biomass	57.04	**0.000**	3.53	**0.033**	3.47	**0.035**
Fungi biomass	1.70	0.195	3.01	0.054	0.77	0.466
The ratio of bacteria biomass to fungi biomass	0.17	0.684	4.47	**0.014**	0.13	0.878
Gram-negative bacteria biomass	1.71	0.371	9.36	**0.000**	3.59	**0.034**
Gram-positive bacteria biomass	6.60	**0.039**	2.75	0.070	7.46	**0.001**
The ratio of gram-positive bacteria to gram-negative bacteria	4.59	0.077	6.21	**0.003**	6.62	**0.002**
Actinomycete biomass	25.08	**0.028**	0.41	0.668	4.91	**0.010**
AMF biomass	30.58	**0.000**	0.69	0.502	0.31	0.732
Protozoa biomass	1.94	0.246	0.20	0.819	2.36	0.102

**Fig 3:**
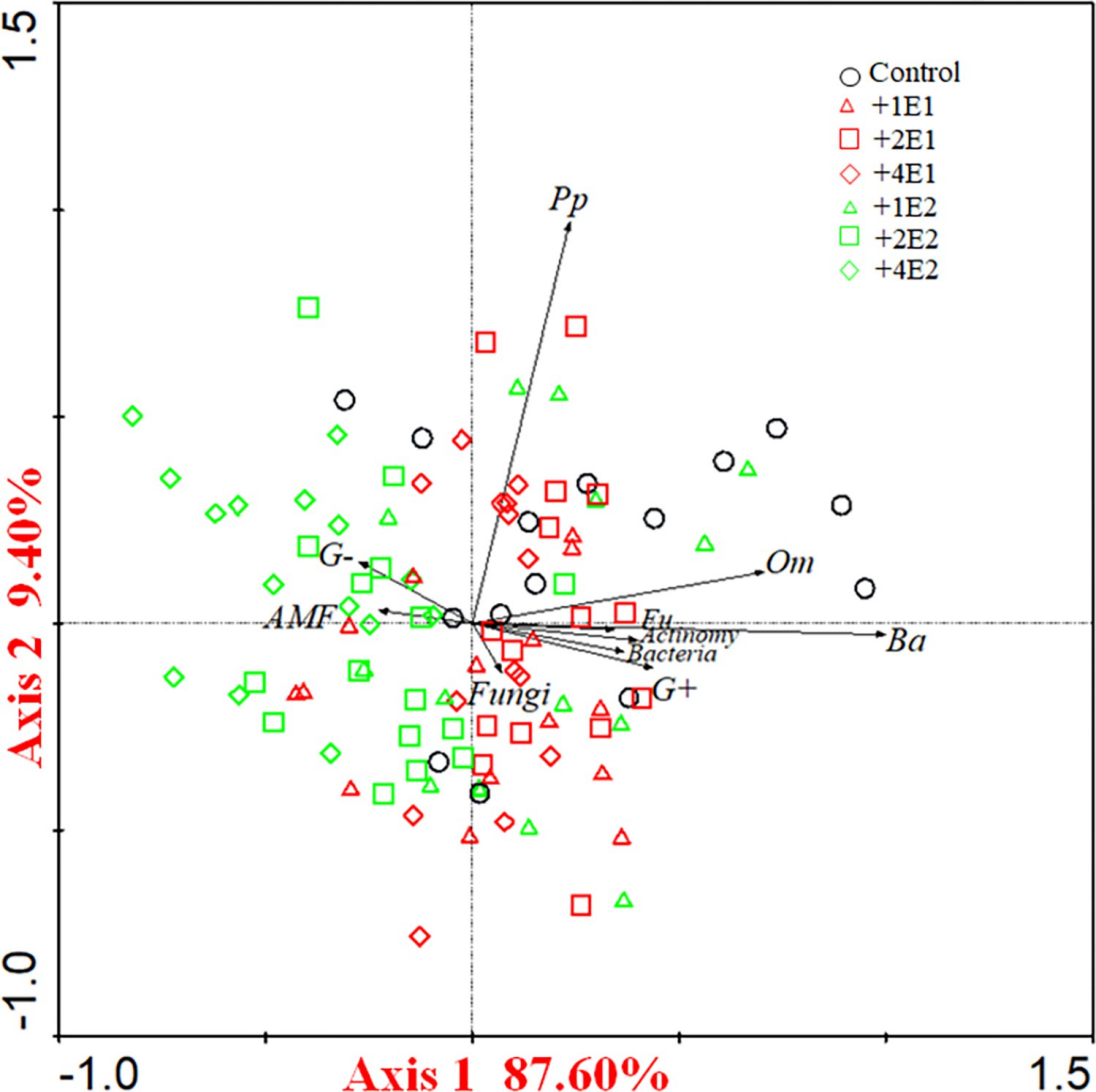
Principle components analysis (PCA) of soil microbial and nematode communities under two earthworm species (*Eisenia fetida*, *Metaphire guillelmi*), and the different density of *Eisenia fetida* and *Metaphire guillelmi* treatments (Ba, bacterivores; Fu, fungivores; Pp, plant parasites; Om, omnivore-predators; Actinomy, actinomycetes; G+, gram-positive bacteria; G-, gram-negative bacteria; AMF, arbuscular mycorrhizal fungi). +1E1, +2E1, and +4E1 represents added 1, 2, and 4 *Eisenia fetida* earthworm individuals, respectively; +1E2, +2E2, and +4E2 represents added 1, 2, and 4 *Metaphire guillelmi* earthworm individuals, respectively.

The results of principal component analysis (PCA) showed that the compositions of microbial communities varied among the different earthworm species treatments along PC1, which accounted for 87.60% of the total variation (Fig 3). Samples in added *Metaphire guillelmi* were dominated by AMF and gram-negative bacteria; whereas, samples in added *Eisenia fetida* were dominated by bacteria, actinomycetes, and gram-positive bacteria.

### Soil nematode communities

**Fig 4:**
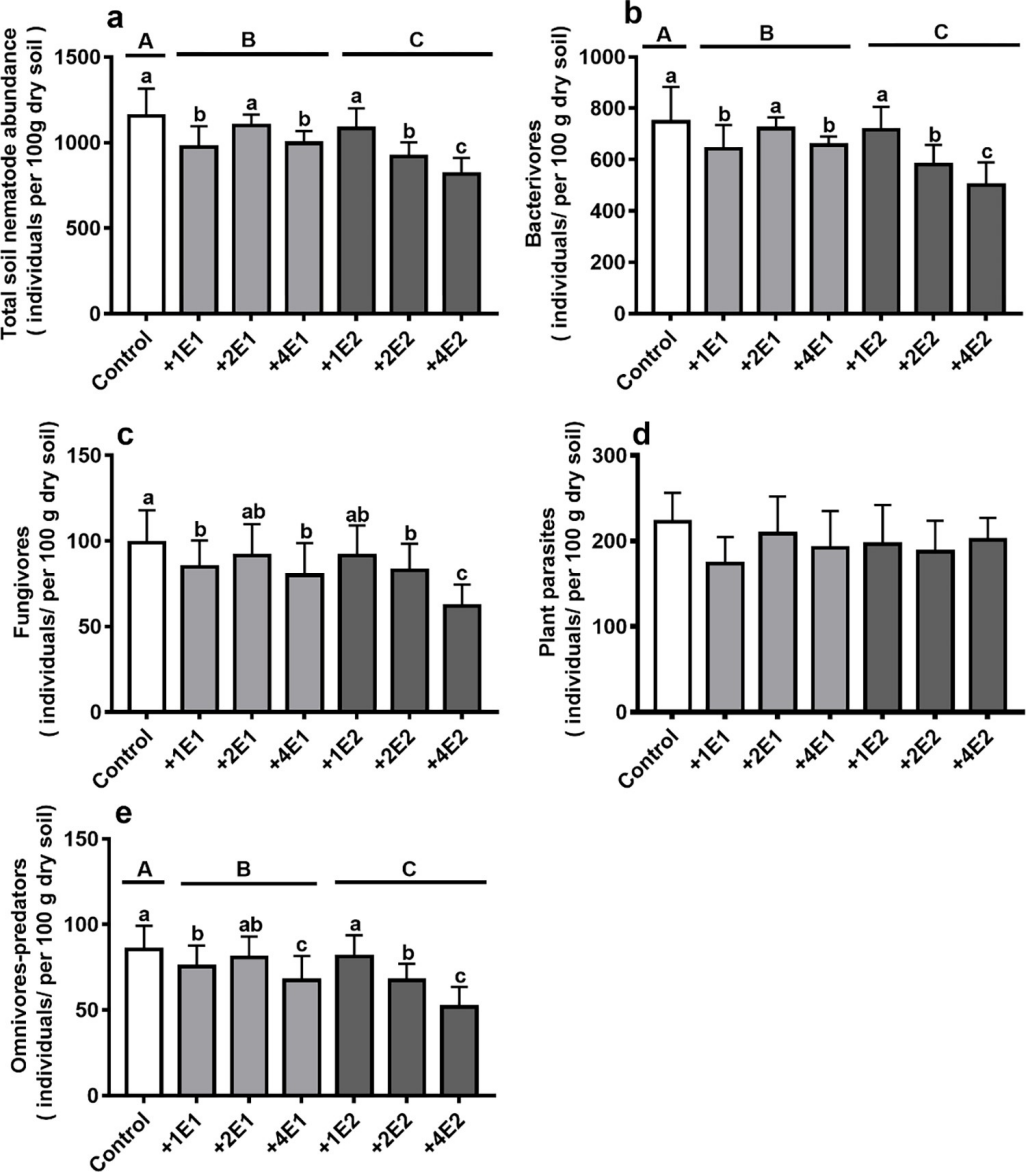
The abundance of soil total nematode (a) and bacterivores (b), fungivores (c), plant parasites (d), and omnivore-predators (e) (individuals / 100 g dry soil weight) under two earthworm species (*Eisenia fetida*, *Metaphire guillelmi*), and the different density of *Eisenia fetida* and *Metaphire guillelmi* treatments. C, control (no added earthworm); +1E, +2E, and +4E represents added 1, 2, and 4 earthworm individuals, respectively. Different uppercase letters indicate significant difference in different earthworm species. Different lowercase letters indicate significant difference in different density under each earthworm species, relative to the control treatments (replicates = 15).

Earthworm species had significant impact on the abundance of total soil nematode, bacteria, and omnivore-predators **(Table 6)**. Compared with the control treatments, added *Eisenia fetida* and *Metaphire guillelmi* treatments significantly decreased the abundance of total soil nematode (Fig 4A), bacterivores (Fig 4B), and omnivore-predators (Fig 4E).

**Table 6:** Results of linear mixed models examining the effects of earthworm species, the different earthworm density addition, and the nested density of earthworm species on soil nematode indexes.

Soil nematode index	Earthworm species		The different earthworm density addition		Earthworm species (earthworm density)	
	F	*P*	F	*P*	F	*P*
Total soil nematode	11.66	**0.001**	11.59	**0.000**	1160	**0.000**
Bacterivores	12.24	**0.001**	10.56	**0.000**	10.09	**0.000**
Fungivores	2.05	0.155	8.88	**0.000**	2.73	0.070
Plant-parasites	0.02	0.896	0.14	0.873	3.93	**0.023**
Omnivore-predators	7.17	**0.009**	16.57	**0.000**	4.70	**0.011**

Different earthworm density significantly affected the abundance of total soil nematode, bacterivores, fungivores, and omnivore-predators (Table 6). Compared with the control treatments, added 1, 4 *Eisenia fetida* earthworm treatments decreased the abundance of total soil nematode (Fig 4A), bacterivores (Fig 4B), and fungivores (Fig 4C), and omnivore-predators (Fig 4E), and added 2, 4 *Metaphire guillelmi* earthworms decreased the abundance of total soil nematode (Fig 4A), bacterivores (Fig 4B), fungivores (Fig 4C), and omnivore-predators (Fig 4E).

The PCA of nematode community also distinguished added *Metaphire guillelmi* and added *Eisenia fetida* along PC1 (Fig 3), with bacterivores and omnivore-predators being dominant in the added *Eisenia fetida* treatments.

### Associations between soil biota and soil parameters

**Fig 5:**
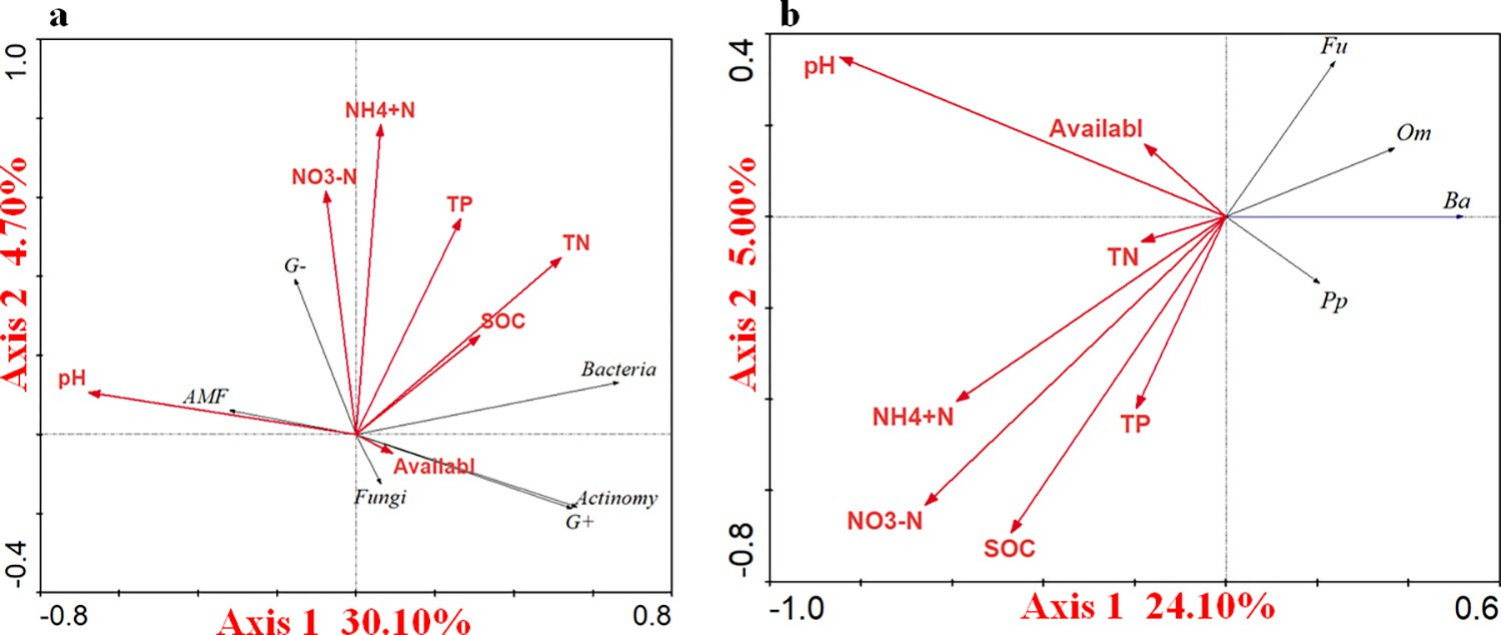
Redundancy analysis (RDA) showing the relationship between soil biota ((a) soil microbial community; (b) nematode community) and soil parameters. NH_4_^+^N, ammonium nitrogen; NO_3_^-^N, nitrate nitrogen; TP, total phosphorus; TN, total nitrogen; SOC, organic carbon; Availabl, available phosphorus. Actinomy, actinomycetes; G+, gram-positive bacteria; G-, gram-negative bacteria; AMF, arbuscular mycorrhizal fungi. Ba, bacterivores; Fu, fungivores; Pp, plant parasites; Om, omnivore-predators.

The RDA analysis suggested that the first axis (F = 41.77, *P* = 0.002) and the second axis (F = 7.58, *P* = 0.002) explained 30.10% and 4.70% of the total variations in soil microorganisms, respectively (Fig 5A). NO_3_^-^N, and NH_4_^+^N were the most important contributors to the distribution of microbial communities (Fig 5A).

The RDA analysis suggested that the first axis (F = 30.82, *P* = 0.002) and the second axis (F = 4.62, *P* = 0.002) explained 24.10% and 5.00% of the total variations in soil nematodes, respectively (Fig 5B). NO_3_^-^N, NH_4_^+^N, and SOC were the most important contributors to the distribution of nematode communities (Fig 5B).

### Effect of earthworm species on the soil micro-food web

**Fig 6:**
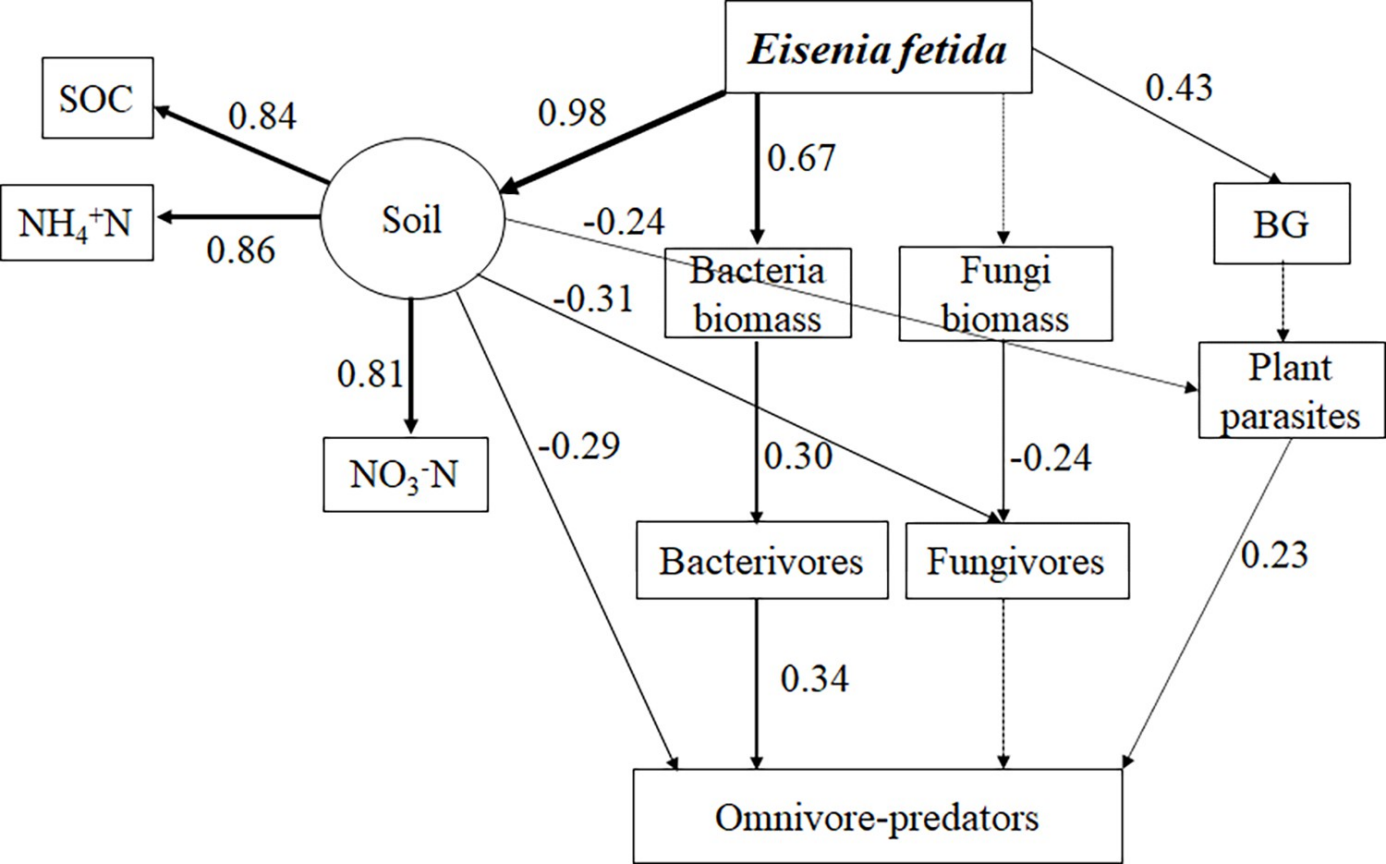
Structural equation models showing the feeding relationship in the food web (soil microorganism, and soil nematode communities) in response to the addition of the different density earthworms (*Eisenia fetida*) (n = 60) (χ^2^ = 43.913, df = 56, *P* = 0.879, CFI = 1.000, GFI = 0.907, RMSEA = 0.000). Numbers on arrows are standardized path coefficients. Solid arrows suggested the effects were significant (P < 0.05) and the thickness represents the magnitude of the path coefficients. Dashed arrows represent the effects were nonsignificant (*P* > 0.05). BG, the belowground biomasses of winter wheat.

**Fig 7:**
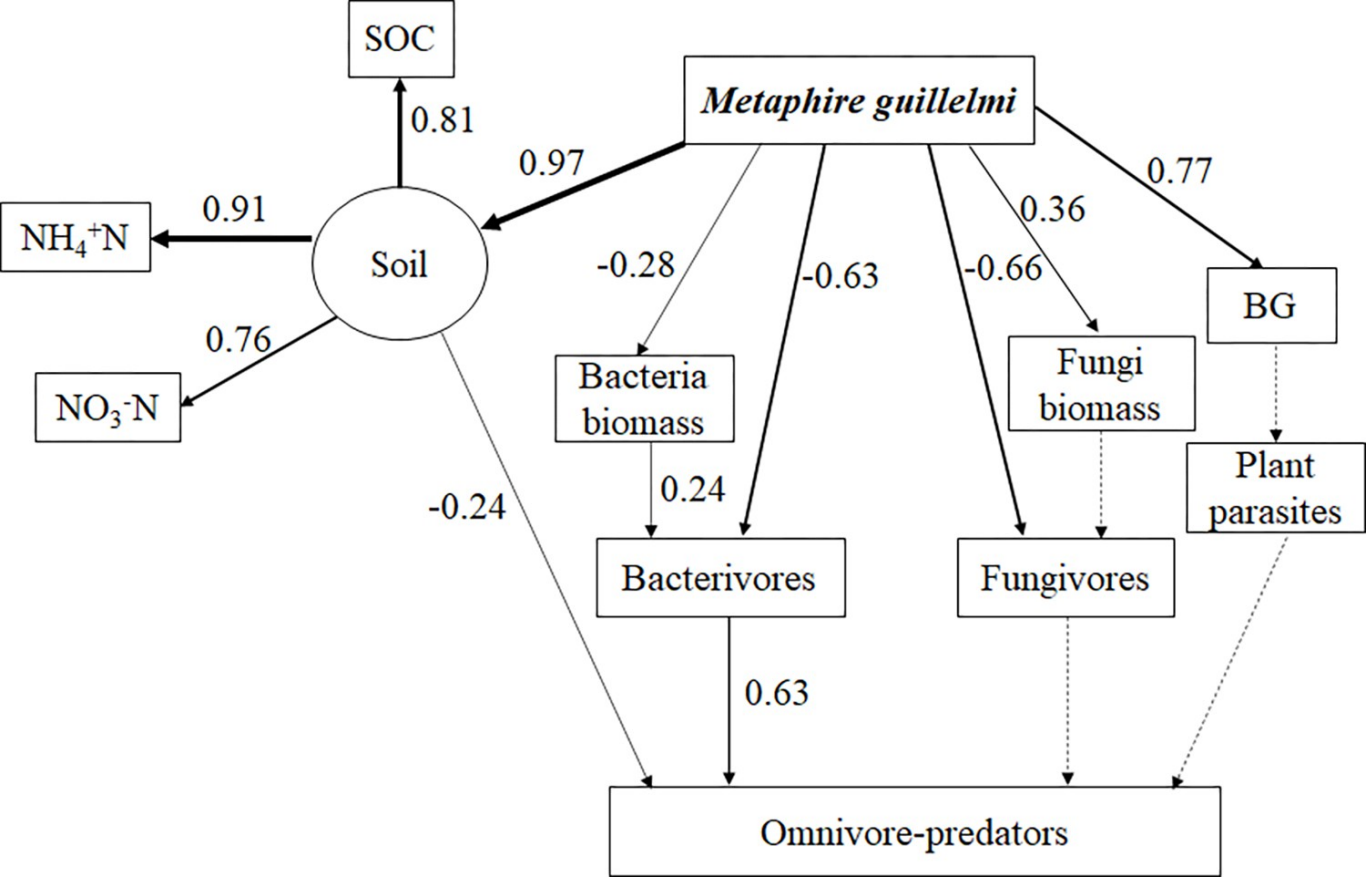
Structural equation models showing the feeding relationship in the food web (soil microorganism, and soil nematode communities) in response to the addition of the different density earthworms (*Metaphire guillelmi*) (n = 60) (χ^2^ = 55.998, df = 64, P = 0.919, CFI = 1.000, GFI = 0.889, RMSEA = 0.000). Numbers on arrows are standardized path coefficients. Solid arrows suggested the effects were significant (P < 0.05) and the thickness represents the magnitude of the path coefficients. Dashed arrows represent the effects were nonsignificant (P > 0.05). BG, the belowground biomasses of winter wheat.

In the present study, SEM was used to examine the effects of the different densities of two earthworm species on soil micro-food web. Overall, the results of our study demonstrated that the central role of the bacterial energy channel, and bottom-up effects dominated in added *Eisenia fetida* (Fig 6) and *Metaphire guillelmi* soil ecosystems (Fig 7).

The addition of *Eisenia fetida* at different density had positive effects on soil physical and chemical properties (SOC, and NH_4_^+^N and NO_3_^-^N), according to SEM (Fig 6). The abundance of fungivores, plant-parasites, and omnivore-predators were negatively influenced by soil SOC, NO_3_^-^N, and NH_4_^+^N. The addition of *Eisenia fetida* with different density directly contributed to the bacteria biomasses, and the belowground biomasses of winter wheat. Bacteria biomass showed bottom-up effects on bacterivores (*P* < 0.05). Bacterivores, and plant parasites both exerted bottom-up effects on omnivore-predators (Fig 6).

The SEM results indicated that the addition of *Metaphire guillelmi* at different density had positive effects on soil properties (NH_4_^+^N, SOC, and NO_3_^-^N), and the belowground biomasses of winter wheat. However, the addition of *Metaphire guillelmi* with different density had negative effects on bacteria biomass, bacterivores, and fungivores (Fig 7). At the same time, soil properties had negative effects on omnivore-predators. Bacteria biomass had bottom-up effects on bacterivores. Omnivore-predators showed a positive correlation with bacterivores (Fig 7).

## Discussion

### Effects of *Eisenia fetida* on soil properties, microbial and nematode communities

The addition of *Eisenia fetida* increased soil organic carbon (SOC). One possible explanation is that earthworm casts contained SOC. The previous study found when compared to the control treatments, SOC increased by 40%-48% on average [44]. Another possible explanation is that the epigeic earthworms accelerated the release of organic substrates by ingestion, fragmentation, and mixing of maize residues with soil [45,46]. The addition of *Eisenia fetida* earthworm at different density led to a strong increase of soil NH_4_^+^N and NO_3_^-^N. The increase in soil NH_4_^+^N and NO_3_^-^N has been found in previous studies [1,3,47]. One reason may be earthworm epidermal mucus containing NH_4_^+^N and NO_3_^-^N [48]; another possible reason may be epigeic earthworms enhancing microbial activity and accelerating nutrient mineralization through casting and burrowing activities [49,50].

We found that added *Eisenia fetida* significantly increased the biomasses of total microorganisms and bacteria. Reason may be due to that *Eisenia fetida*, as detritus organism, mainly feed on soil organic matter (maize straw), few soil and little soil microorganisms. After the addition of maize straw, carbon, nitrogen, phosphorus, and potassium in maize straw improved the activities and quantities of soil microorganisms. In addition, *Eisenia fetida* increased the surface area of microbial erosion during feeding the crushed maize straw process [18], resulting in the increasing quantities of soil microorganisms. At the same time, *Eisenia fetida* secreted mucus, and excreted casts, indirectly stimulating soil microorganisms. Actinomycetes, and gram-positive bacteria were dominant in the added *Eisenia fetida* treatments. This may be due to that neutral pH and appropriate temperature and humidity in the intestine of *Eisenia fetida*, which are conducive to the survival of actinomycetes, and gram-positive bacteria [51].

Compared with the control treatments, *Eisenia fetida* addition significantly decreased the abundance of bacterivores, and omnivore-predators. Reason may due to that *Eisenia fetida* directly ingest soil nematodes through biotic factor such as predating [16,52], or decrease soil nematodes by the proteolytic activity of enzymes present in the gut contents of earthworms [53].

### Effects of the different density *Eisenia fetida* addition on the soil micro-food web

Our study demonstrated that the addition of *Eisenia fetida* resulted in a bacterial-dominated energy channel, which was accordance with the previous study [3,54]. There was no significant trophic interaction of omnivore-predators feeding on bacterivores, fungivores and plant parasites in our study. *Eisenia fetida* addition negatively affected the abundance of fungivores, plant parasites and omnivore-predators by increasing the content of soil SOC, NO_3_^-^N, and NH_4_^+^N (Fig 6). RDA analysis demonstrated that SOC, NO_3_^-^N, and NH_4_^+^N had negative effects on fungivores, plant parasites and omnivore-predators (Fig 5B). Reason may be due to that NO_3_^-^N, and NH_4_^+^N potentially had negative impacts on soil nematodes [55]. There was bottom-up control plant parasites prey to omnivore-predators, which resulted in the increase in the abundance of plant parasites. However, *Eisenia fetida* had negative effect on plant parasites indirectly by changing soil properties. Therefore, there was no significant change observed in plant parasites.

One of the most important issues in ecology is whether the soil micro-food webs are regulated by resources (bottom-up controlled) or by predators (top-down controlled) [56]. Our study demonstrated that the bottom-up effects were more important than the top-down forces for the structure of the soil micro-food web in our study, indicating that the indirect effects were more important than direct effects under the addition of *Eisenia fetida* at different density treatments, which was accordance with our first hypothesis. Although the biomass of protozoa indicated by PLFA biomarkers was tested, protozoa were not included in the statistical analyses of this study, which mainly was due to relatively low biomass. Notably, protozoa play decisive roles in soil and should be considered in future studies.

### Effects of *Metaphire guillelmi* on soil properties, microbial and nematode communities

Previous studies demonstrated that the anecic earthworms *Lumbricus terrestris* and *Aporrectodea longa* increased the content of soil organic carbon by 4.1–21.0%, and 21.2–43.0% by burrow-wall material and casts, respectively [57]. In our study, the addition of *Metaphire guillelmi* with different densities increased SOC of the pot soil which was consistent with the previous findings. The increase of NH_4_^+^N and NO_3_^-^N were mainly due to *Metaphire guillelmi* facilitated soil N mineralization [58,59], and the rise in soil mineral N has been found in the previous experiments [1,47].

Our results indicated that added *Metaphire guillelmi* significantly decreased the biomasses of total microorganisms, bacteria, gram-positive bacteria, and actinomycetes. One reason may due to that *Metaphire guillelmi* have a bigger body than *Eisenia fetida* (Table 2), and therefore consume a large amount of soil. This resulted in the reduction of soil microorganisms. Another reason may be attributed to the fact that *Metaphire guillelmi* is capable of secreting a large amount substances that inhibit the quantity of soil microorganisms [60]. Gram-negative bacteria, and AMF were dominant in the added *Metaphire guillelmi* treatments. This may be because gram-negative bacteria possess an outer membrane composed of lipopolysaccharide to protect them from certain types of chemical attack [61]. The increase in AMF might be attributed to two reasons. One reason is that *Metaphire guillelmi* possesses a strong bio-disturbance ability, which makes it more conducive to promoting the formation of soil aggregates during the feeding process. This indirectly helps the protection of soil mycorrhizal fungi. Additionally, the body surface of the *Metaphire guillelmi* or around the wormhole contains numerous mycorrhizal propagules. Furthermore, the hormone substances in *Metaphire guillelmi*’ casts can facilitate mycorrhizal infection [62].

The introduction of *Metaphire guillelmi* led to significant decrease in the abundance of bacterivores, and omnivore-predators. Previous study demonstrated that *Metaphire guillelmi* significantly decreased the abundance of bacterivores [63]. The reduction in nematode populations may be associated with earthworm gut passage [64]. The decrease of omnivore-predators was attributed to that the competition for food between *Metphire guillelmi* and omnivore-predators.

### Effects of the different density *Metaphire guillelmi* addition on the soil micro-food web

A bacterial-dominated energy channel was found in the addition of *Metaphire guillelmi* treatments. Bottom-up effects were dominant in the soil micro-food web, which was accordance with our first hypothesis. The addition of *Metaphire guillelmi* with different densities increased the belowground biomasses of winter wheat (Fig 1B), which can provide more rhizosphere-deposited C to the microbial community, and improved the bottom-up effects. Bacterivores had bottom-up effects on omnivore-predators, resulting in an increase in the abundance of omnivore-predators. However, we speculated that the negative effects of *Metaphire guillelmi* on omnivore-predators by indirectly changing soil properties (Fig 5B) were in a dominant position. RDA analysis results also demonstrated soil properties had negative effects on omnivore-predators (Fig 5B). Therefore, in our study, added *Metphire guillelmi* decreased the abundance of omnivore-predators.

In summary, the responses of the soil micro-food web to the addition of *Eisenia fetida* and *Metaphire guillelmi* at different densities may depend on the earthworm species, which demonstrated our second hypothesis. As earthworms are the most important soil biota, we attempted to explain the effect of earthworms on the soil micro-food web structure. These explanations were not complete enough. Firstly, earthworms interact with soil habitat, soil microorganisms, soil nematodes in various ways. For example, they can change the habitat of soil microorganisms and soil nematodes, act as the feeders of soil microbial and nematode, and disperse soil microorganisms and nematodes. These interactions can potentially affect the effect of earthworms on the soil micro-food web. Secondly, other groups of soil fauna, such as enchytraeids and microarthropods, frequently play decisive functional roles in soils and should be considered in future research. Thirdly, it is important to consider long-term field experiments that examine the impact of various earthworm species on the soil micro-food web. As a result, further field research is necessary to understand the mechanism of different earthworm species on the soil micro-food web.

## Conclusions

In the present study, our results show that the bottom-up effects are dominant and identify the central role of bacterial energy pathways in the soil food webs under two earthworm species, and different densities of two earthworm species treatments. The addition of *Eisenia fetida* at different density had negative effects on the abundance of omnivore-predators indirectly by changing soil properties. However, the addition of *Metaphire guillelmi* at different density had a direct negative impact on bacterivores, and fungivores, but had an indirect negative effect on omnivore-predators by altering soil properties. The comprehensive effects of *Metaphire guillelmi*, which include both direct and indirect interaction, may significantly impact the structure of the soil micro-food web. Overall, SEM provides complex interactions and energy channels in our soil micro-food webs, which will facilitate a better mechanistic understanding within soil food webs. However, our results are solely based on the experiment done in an artificial indoor environment. Notably, field environment is quite complex, soil fauna including protozoa, micro and meso-fauna, spider, and ground beetles affect directly or indirectly the soil micro-food web. Therefore, more field study, including other groups of soil fauna, such as protozoa, micro- and meso-arthropod, and so on, are needed to explain the effects of the different earthworm species on the soil micro-food web.

## Supporting information

S1 Fig The aboveground and belowground biomasses (dry weight g) of winter wheat under two earthworm species (*Eisenia fetida*, *Metaphire guillelmi*), and the different density of *Eisenia fetida* and *Metaphire guillelmi* treatments. (XLSX)

S2 Fig The biomasses of total soil microorganism, bacteria, fungi, G- bacteria, G+ bacteria, Actinomycetes, AMF, and Protozoa under two earthworm species (*Eisenia fetida*, *Metaphire guillelmi*), and the different density of *Eisenia fetida* and *Metaphire guillelmi* treatments. G- bacteria, gram-negative bacteria; G+ bacteria, gram-positive bacteria; AMF, arbuscular mycorrhizal fungi.
(XLSX)

S3 Fig The relative biomasses of bacteria, fungi, AMF, G-, G+, and actinomycetes and the abundance of Ba, Fu, Pp, and Om under two earthworm species (*Eisenia fetida*, *Metaphire guillelmi*), and the different density of *Eisenia fetida* and *Metaphire guillelmi* treatments. AMF, arbuscular mycorrhizal fungi; G-, gram-negative bacteria; G+, gram-positive bacteria; Ba, bacterivores; Fu, fungivores; Pp, plant parasites; Om, omnivore-predators.
(XLSX)

S4 Fig The abundance of total soil nematode, bacterivores, fungivores, plant-parasites, and omnivore-predators under two earthworm species (*Eisenia fetida*, *Metaphire guillelmi*), and the different density of *Eisenia fetida* and *Metaphire guillelmi* treatments. (XLSX)

S5 Fig The soil microorganisms, soil nematodes, and soil physicochemical properties under two earthworm species (*Eisenia fetida*, *Metaphire guillelmi*), and the different density of *Eisenia fetida* and *Metaphire guillelmi* treatments. (XLSX)

S6 Fig SOC, NO3-N, NH4+N, and microbial bacteria biomass, fungi biomass, and the abundance of Ba, Fu, Pp, and Om, the belowground biomasses of winter wheat under the different density of *Eisenia fetida* treatments. SOC, organic carbon; NO_3_^-^N, nitrate nitrogen; and NH_4_^+^N, ammonium nitrogen. Ba, bacterivores; Fu, fungivores; Pp, plant parasites; Om, omnivore-predators.
(XLSX)

S7 Fig SOC, NO3-N, NH4+N, and microbial bacteria biomass, fungi biomass, and the abundance of Ba, Fu, Pp, and Om, the belowground biomasses of winter wheat under the different density of *Metaphire guillelmi* treatments. SOC, organic carbon; NO_3_^-^N, nitrate nitrogen; and NH_4_^+^N, ammonium nitrogen. Ba, bacterivores; Fu, fungivores; Pp, plant parasites; Om, omnivore-predators.
(XLSX)

S1 Table The Earthworm biomasses at the beginning and end of experiment under two earthworm species (*Eisenia fetida*, *Metaphire guillelmi*), and the different density of *Eisenia fetida* and *Metaphire guillelmi* treatments. (XLSX)

S2 Table The body length (cm), width (cm), Gizzard diameter (cm), Foregut length (cm), Midgut length (cm), and Hindgut length (cm) of two earthworm species (*Eisenia fetida*, *Metaphire guillelmi*). (XLSX)

S3 Table The physical and chemical properties (TC, TN, TP, SOC, available P, NO3-N, NH4+N, pH) and the aboveground and belowground biomasses of winter wheat under two earthworm species (*Eisenia fetida*, *Metaphire guillelmi*), and the different density of *Eisenia fetida* and *Metaphire guillelmi* treatments. TC, Total carbon; TN, total nitrogen; TP, total phosphorus; SOC, organic carbon; available P, available phosphorus; NO_3_^-^N, nitrate nitrogen; and NH_4_^+^N, ammonium nitrogen.
(XLSX)

S4 Table Soil physical and chemical properties under the different density *Eisenia fetida* and *Metaphire guillelmi* addition treatments. (XLSX)

S5 Table The biomass of soil total microorganism, bacteria, fungi, gram-negative bacteria, gram-positive bacteria, actinomycetes, AMF (arbuscular mycorrhizal fungi), and protozoa, the ratio of bacteria biomass to fungi biomass, the ratio of gram-positive bacteria to gram-negative bacteria under two earthworm species (*Eisenia fetida*, *Metaphire guillelmi*), and the different density of *Eisenia fetida* and *Metaphire guillelmi* treatments. (XLSX)

S6 Table The abundance of total soil nematode, bacterivores, fungivores, plant-parasites, and omnivore-predators under two earthworm species (*Eisenia fetida*, *Metaphire guillelmi*), and the different density of *Eisenia fetida* and *Metaphire guillelmi* treatments. (XLSX)
